# Performance of galactomannan testing from endotracheal aspirate to guide bronchoalveolar lavage in the diagnosis of invasive aspergillosis

**DOI:** 10.1007/s15010-023-01985-1

**Published:** 2023-02-04

**Authors:** Karl Dichtl, Rachel Barry, Matthias W. A. Angstwurm, Sebastian Suerbaum, Johannes Wagener

**Affiliations:** 1 Max von Pettenkofer-Institut für Hygiene und Medizinische Mikrobiologie, Medizinische Fakultät, LMU München, Munich, Germany; 2 Diagnostic and Research Institute of Hygiene, Microbiology and Environmental Medicine, Medical University of Graz, Graz, Austria; 3 Microbiology Department, St. James’s Hospital, Dublin, Ireland; 4 Medizinische Klinik und Poliklinik IV, LMU Klinikum, Munich, Germany; 5 Department of Clinical Microbiology, School of Medicine, Trinity College Dublin, the University of Dublin, St. James’s Hospital Campus, Dublin, Ireland

**Keywords:** Invasive aspergillosis, Galactomannan, Bronchoscopy, BAL, Endotracheal aspirate

## Abstract

**Purpose:**

Invasive aspergillosis is a major threat to immunocompromised individuals. Galactomannan (GM) is used as a biomarker for invasive aspergillosis. Investigations recommended in current guidelines include GM testing of bronchoalveolar lavage (BAL) fluids. GM testing of endotracheal aspirate, the sampling of which is less invasive, less resource-intensive and less aerosol-generating, is not validated. We compared the performance of endotracheal aspirate GM as a screening tool to predict BAL fluid GM-positivity in patients with suspected invasive aspergillosis.

**Methods:**

Of each patient, a pair of corresponding endotracheal aspirate and BAL fluid samples was tested and compared for GM results. Two sample sets were included. The first consisted of 140 consecutive BAL fluid/endotracheal aspirate pairs obtained from 133 patients. The pairs of the second sample set (*n = *38) were selected based on the criterion that the BAL tested positive for GM. All specimens were obtained in a German 2,000 bed tertiary care center.

**Results:**

Among BAL fluid GM-positive samples, endotracheal aspirate GM demonstrated poor specificity (72%) but high sensitivity (92% in predicting BAL fluid GM of ≥ 0.50 and 91% for BAL fluid GM of ≥ 1.00) and an excellent negative predictive value (98%). The use of a marginally elevated cutoff of 0.63 resulted in an improved specificity (72–81%), without loss of sensitivity.

**Conclusions:**

For screening purposes, one might consider testing endotracheal aspirate for GM, which could help avoid unnecessary BAL.

## Introduction

Invasive aspergillosis (IA) is an invasive fungal infection caused by *Aspergillus* species and poses a major threat to immunocompromised individuals. Diagnosis is challenging, owing to the pathogen’s ubiquitous nature and non-specific clinical presentation, and is therefore based on a combination of clinical, microbiological, and radiological findings [1]. Early diagnosis allowing targeted and timely antifungal chemotherapy is a crucial determinant for survival. Galactomannan (GM), a fungal cell wall polysaccharide expressed by *Aspergillus* hyphae, can be detected in blood and bronchoalveolar lavage (BAL) fluid of patients with IA and is a well-established diagnostic tool, overcoming the limitations of culture from respiratory tract samples, including low sensitivity in pretreated patients and an overall low specificity [1].

A significant disadvantage of GM testing on sera is reduced sensitivity in specific settings, i.e., in patients receiving mold-active prophylaxis [2], and in non-neutropenic patients, such as solid organ transplant recipients [3] or intensive care unit (ICU) patients [4]. In such high-risk patients, GM testing in BAL fluid is more reliable in terms of sensitivity and hence is recommended as a preferential tool, with excellent diagnostic performance [5]. It should be noted, however, that bronchoscopy is a resource-intensive investigation, which often hampers its routine application. Furthermore, the patient is exposed to an invasive procedure with an average complication rate of 10%, including major adverse events, such as arrhythmias, severe bleeding, and requirement for prolonged mechanical ventilation or vasopressor support [6]. Hence, it is comprehensible that clinicians attempt to avoid BAL when possible, opting for less invasive sampling of respiratory tract fluids.

However, the use of GM on alternative respiratory specimens, including sputum or endotracheal aspirate (ETA), has not been validated. The aim of this study was to evaluate the performance of ETA GM as a screening tool to predict BAL GM-positivity.

## Methods

The analysis was performed at the Max von Pettenkofer-Institute for Hygiene and Medical Microbiology. All samples were obtained in the University Hospital of Ludwig-Maximilians-University, a 2000-bed tertiary care center in Munich, Germany. We retrospectively identified two sets of BAL and corresponding ETA pairs of which at least the BAL was primarily sampled for GM testing. The first set consisted of 140 consecutive BAL/ETA pairs obtained from 133 patients, with a maximum time difference of one day between ETA and BAL sampling. This collection included all pairs between January 2017 and April 2020. To strengthen the significance of our analysis, an additional second BAL/ETA set (*n = *38) was analyzed. All those pairs were retrospectively selected on the criterion that the BAL tested positive for GM. Here, all available pairs with a time difference of up to one week and sampling dates from 2012 to 2020 were included. Patient characteristics are summarized in Table 1 and sample characteristics in Table 2.

**Table 1:** Clinical and microbiology data and demographic characteristics of all included cases (A) and of the cases with GM-positive (pos.) BAL results (B)

(A)	All patients		Consecutive cohort^a^	
	*n*	(%)	*n*	(%)
Number	171	(100)	133	(100)
Age				
Median	57	–	56	–
Range	20–84		20–84	
Sex				
Female	64	(37)	58	(44)
Male	107	(63)	75	(56)
Number of specimens per patient				
Patients with one BAL/ETA pair	164	(96)	126	(95)
Patients with two BAL/ETA pairs	7	(4)	7	(5)
Treated in intensive care unit	107	(63)	75	(56)

(B)	All BAL GM-pos. patients		BAL GM-pos. patients in consecutive cohort	
	*n*	(%)	*n*	(%)
Number	61	(100)	22	(100)
EORTC/MSGERC criteria^b^				
Category				
Proven	4	(7)	1	(5)
Probable	19	(31)	7	(32)
Known host factors	28	(46)	12	(55)
Mycological evidence apart from the respective BAL	41	(67)	18	(82)

^a^The consecutive cohort includes all consecutive BAL/ETA pairs (max. distance of corresponding BAL and ETA sampling dates: one day) that were obtained in a 40-month period

^b^Evaluation according to the EORTC/MSGERC criteria [7] was performed for all individuals with positive BAL GM. Percentages in parentheses

**Table 2:** Comparison of BAL and ETA sampling dates (A) and their influence on ETA GM sensitivity for the prediction of BAL GM-positivity (B)

	All samples						Consecutive samples		
(A) Characteristics of BAL/ETA pairs									
Total number	178		(100)		140			(100)	
BAL sampled before ETA	39		(22)			18		(13)	
Mean distance in days		3	–			1			–
Median distance in days		2	–			1			–
BAL and ETA sampled on same day	104		(58)		97			(69)	
BAL sampled after ETA	35		(20)		25			(18)	
Mean distance in days		3		–	1				–
Median distance in days		1		–	1				–

	ETA GM positivity in BAL GM positive ETA/BAL pairs			
	Consecutive cohort		Additional sample set^a^	
(B) Sensitivity of ETA GM for prediction of BAL GM-positivity depending on sampling dates (max. distance between BAL and ETA sampling date)				
± 7 days	n/a		30/38	(79)
± 3 days	n/a		19/24	(79)
± 2 days	n/a		12/13	(92)
± 1 day	22/24	(92)	9/9	(100)

Percentages in parentheses. ETA GM positivity was defined as a GM result ≥ 0.5

^a^While the consecutive cohort includes all consecutive BAL/ETA pairs that were obtained in a 40-month period, the additional sample set includes exclusively BAL/ETA pairs with GM-positive BALs, which were identified in a retrospective review. n/a, not applicable (inclusion criterion for consecutive cohort: maximal distance between BAL and ETA sampling date of one day)

All measurements were performed using the Platelia *Aspergillus* enzyme-linked immunosorbent assay (EIA) (Bio-Rad Laboratories, Marnes-la-Coquette, France) following the manufacturer’s instructions for processing BAL fluid. A positive GM was defined as an index value of ≥ 0.50 as recommended by the manufacturer for the interpretation of BAL fluid. Neither the type of sample (ETA vs. BAL) nor the association of one sample to a specific BAL/ETA pair nor the result of the other sample of the pair, were known to the persons performing or evaluating the EIA.

For all BAL GM-positive cases, we performed a retrospective chart review and subcategorized these patients into proven, probable, and no IA, according to the consensus definitions of invasive fungal diseases of the European Organization for Research and Treatment of Cancer and the Mycoses Study Group Education and Research Consortium (EORTC/MSGERC) [7]. None of the respective BAL/ETA pairs contained molds other than *Aspergillus* species.

Statistical analysis was performed using GraphPad Prism 5 (GraphPad Software, San Diego, USA).

## Results

**Fig. 1:**
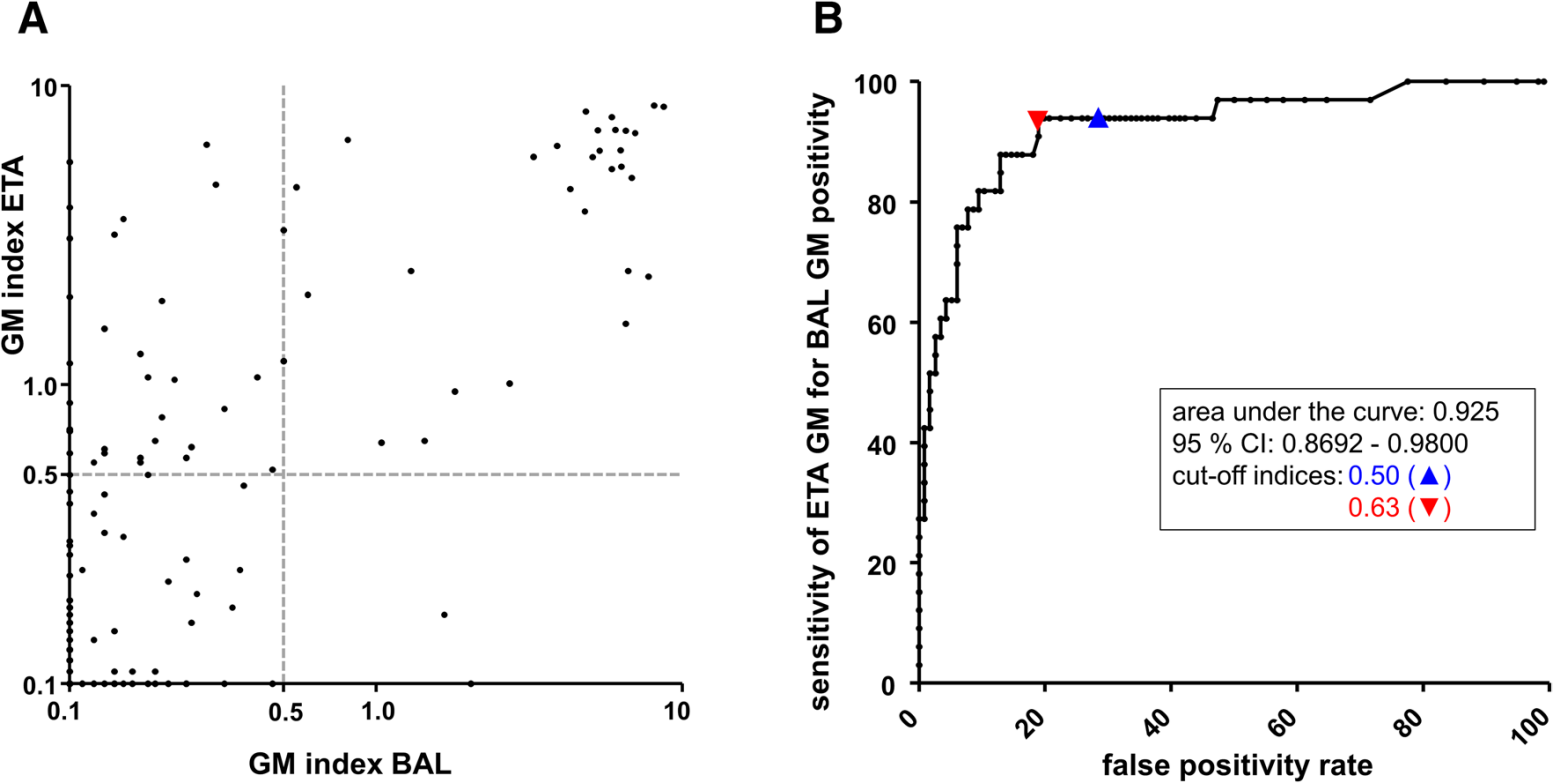
**A** Results of galactomannan (GM) testing from endotracheal aspirate (ETA) and bronchoalveolar lavage (BAL) fluid. Dotted lines indicate the cutoff of the GM ELISA as recommended by the manufacturer for serum and BAL samples. For better visualization, all results < 0.10 were plotted at 0.1. **B** ROC curve analysis of ETA GM testing for the prediction of BAL GM positivity. Both graphs include all BAL / ETA pairs with a maximum time difference of one day between the sampling dates. CI, confidence interval

Of the 140 consecutive BAL/ETA pairs included in this study, BAL GM was positive in 17% of cases, versus 39% of ETA samples. While overall BAL/ETA results correlated poorly (Spearman’s correlation coefficient: 0.49), BAL/ETA correlation in BAL-positive cases was encouraging (Fig. 1A). Twenty-two of 24 BAL GM-positive sample pairs were ETA GM-positive, resulting in an ETA sensitivity for BAL GM-positivity of 92% (confidence interval [CI]: 74–99%; Table 3). Additionally, ETA GM-negativity was found to be a promising predictor of BAL GM-negativity: eighty-three of 85 ETA GM-negative correlated with BAL GM-negativity, resulting in a negative predictive value (NPV) for BAL GM of 98% (CI 92–100%). Specificity and positive predictive value (PPV) for BAL GM of ETA were only 72% (CI 63–79%) and 40% (CI 28–53%), respectively.

**Table 3:** Contingency table for GM results from BAL and ETA samples of the consecutive cohort

Specimen	BAL	
	pos	neg
ETA		
pos	22	33
neg	2	83

To substantiate the hypothesis that ETA GM might be a useful screening marker for lower airway GM-positivity, an additional 38 pairs were included, where BAL GM was known to be positive. Overall, the ETA-positivity rate was only 79% (30/38 BAL/ETA pairs; CI 64–89%). However, four of the 8 discordant pairs yielded GM amounts just beneath the cut-off of 0.50 (0.40–0.49). As stated, timing between sampling was up to seven days in this cohort (Table 2). Sensitivity increased with closer proximity of BAL/ETA sampling dates (Table 2): 79% for ± 3 days (*n = *24; CI 60–91%), 92% for ± 2 days (*n = *13; CI 67–100%), and finally 100% for ± 1 day (*n = *9; CI 70–100%), the latter of which meets the temporal inclusion criterion of the original set.

All BAL-positive cases (*n = *61, with a total 62 BAL/ETA pairs) were retrospectively reviewed and categorized according to the EORTC/MSGERC definitions for IA [7]. Four and 19 BAL/ETA pairs were obtained from patients with proven and probable IA. Probable IA cases were identified based on the growth of *Aspergillus* from respiratory specimens and/or GM seropositivity (≥ 1.00) and/or positive GM results (≥ 1.00) from an additional, independent BAL sample. Thirty-nine cases did not meet the criteria for proven, probable, or possible IA. However, 31 of those 39 cases did just not meet the EORTC/MEGERC criteria of a host factor, which excluded them from categorization into probable or possible IA, regardless of clinical, microbiology, and radiological findings. ETA GM sensitivity in the subgroup of proven/probable BAL GM-positive cases was 91% (CI 73–98%).

Since there is no recommendation for a GM cut-off in ETA, ROC curve analysis was performed using the results of all pairs with a maximum sampling difference of ± 1 day (area under the curve: 0.925 with CI 0.869–0.980; Fig. 1B). A cut-off of 0.63 yielded the maximum Youden’s index of 0.75. When applied to the set of consecutive BAL/ETA pairs, this cut-off did not result in any loss of sensitivity for BAL GM-positivity (still 92%; CI 74–99%) and produced an increase in both specificity (72–81%; CI 73–87%) and PPV (40% to 50%; CI 36–64%). Furthermore, we found that ETA GM-positivity demonstrated 91% sensitivity (CI 73–98%) in predicting a BAL GM result of ≥ 1.0, a widely proposed cut-off for respiratory samples [8].

To address the risk of bias, sample timing was analyzed to rule out the possibility that a positive ETA result had triggered a subsequent generation of the, respectively, paired BAL. However, in only 10/178 BAL/ETA pairs, results of ETA GM testing were known to the attending physicians before bronchoscopy was performed.

## Discussion

To our knowledge, this is the first study evaluating the concordance of GM in paired BAL/ETA samples in the diagnosis of IA. Previous studies evaluating sputum are limited, and its diagnostic utility remains unclear [9].

ETA GM-positivity had a sensitivity of 92% in predicting a BAL GM of ≥ 0.50 and 91% for a BAL GM of ≥ 1.00. Furthermore, an analysis of a subset of BAL-positive cases demonstrated increasing sensitivity with closer proximity of sampling (79% ± 3 days, up to 100% ± 1 day).

ETA GM is limited by its moderately low specificity and has not been validated. Notably, screening with ETA GM was associated with an excellent NPV for BAL GM positivity (98%). Our findings suggest the use of a marginally elevated cut-off of 0.63, which corresponds with an improved specificity (72% to 81%), without loss of sensitivity. The high test performance of ETA GM for predicting BAL GM positivity raises the question as to whether ETA GM could be used as a screening tool; however, these findings should be confirmed in further studies. In the context of a negative ETA GM result, the attending physicians should consider whether bronchoscopic IA diagnostics is beneficial, given the low pretest probability. A negative ETA GM result may also provide reassurance in cases where bronchoscopy is not possible, e.g., in those who are difficult to ventilate or where there is lack of consent. However, it should also be stated that bronchoscopy may not be pursued for IA diagnostics alone and may be helpful in diagnosing other infections.

Of note, clinical categorization of many BAL-positive cases was limited by strict host factors as set out by the EORTC/MSGERC criteria, e.g., neutropenia with < 0.5*10^9 neutrophils / L for > 10 days or use of ≥ 0.3 mg/kg corticosteroids for ≥ 3 weeks [7]. Subsequently, non-traditional host factors associated with IA have been identified in the literature, including diabetes, ICU stay and mechanical ventilation, as well as immunosuppressive therapy including glucocorticosteroids [10]. Interestingly, consensus definitions for COVID-19-associated aspergillosis (CAPA) state that a microbiological diagnosis of possible IA can be supported by “non-bronchoscopic” lavage, including GM EIA [11]. Although limited, our analysis suggests a high rate of false positivity (39% ETA GM-positive vs. 17% BAL GM-positive) and would pose the question as to whether non-bronchoscopic methods should be relied on for epidemiological definitions.

There are important limitations to our study. Firstly, it is a single-center retrospective study with a very specific aim: to evaluate the performance of ETA GM analysis in predicting BAL GM positivity. Interpreting the results, it must be considered that ETA GM was compared to the results of a widely used surrogate marker but not to presence or absence of infection, i.e., pulmonary IA. Secondly, the reasons why BAL was performed in each pair were not known to the research team. For example, testing could have been triggered by culture results positive for *Aspergillus* or other positive historic GM results from serum, BAL or even ETA, which were outside of the scope of the inclusion criteria for this study, but which would represent important confounders. Thirdly, it is possible, due to the limited insights into patient characteristics, that positive ETA or BAL GM results might represent to a certain extent false-positive results, e.g., due to the introduction of GM antigen into the respiratory tract from the environment, through air- or food-borne contamination in the absence of viable *Aspergillus* conidia [12]. However, the finding of a similar sensitivity in cases of proven / probable IA argues against this limitation. Finally, from the laboratory's point of view, it must be further noted that ETA is an inconvenient specimen for GM testing due to the higher viscosity compared to BAL fluid.

Measuring ETA GM may allow clinical decision makers to avoid bronchoscopy in certain patients. Conceivably, screening with ETA could preclude the need for an invasive intervention which poses an infection control risk to healthcare staff and is associated with a 10% adverse complication rate [6]. This would result in both economic savings and preservation of vital resources within hospitals. However, due to the limitations to our study, the findings should be confirmed in further independent prospective studies assessing GM EIA performance in ETA samples.

## Data Availability

The data that support the findings of this study are available upon reasonable request.
